# Complete Genome Sequence of Neisseria musculi Using Illumina and PacBio Sequencing

**DOI:** 10.1128/MRA.00452-21

**Published:** 2021-06-10

**Authors:** Eliza Thapa, Jain Aluvathingal, Suvarna Nadendla, Aditya Mehta, Hervé Tettelin, Nathan J. Weyand

**Affiliations:** a Department of Biological Sciences, Ohio University, Athens, Ohio, USA; b Institute for Genome Sciences (IGS), University of Maryland School of Medicine, Baltimore, Maryland, USA; c Department of Microbiology and Immunology, IGS, University of Maryland School of Medicine, Baltimore, Maryland, USA; d The Infectious and Tropical Disease Institute, Ohio University, Athens, Ohio, USA; e Molecular and Cellular Biology Program, Ohio University, Athens, Ohio, USA; University of Delaware

## Abstract

Neisseria musculi is an oral commensal of wild-caught mice. Here, we report the complete genome sequence of *N. musculi* strain NW831, generated using a combination of the Illumina and PacBio platforms.

## ANNOUNCEMENT

Animal models that mimic human *Neisseria* infections and persistence are often compromised by strict tropism of pathogenic species for the human host (1). Using *Neisseria* species indigenous to an animal host can avoid host restriction barriers. Neisseria musculi is an oral commensal of healthy wild-caught mice (2). *N. musculi* can colonize the oral cavity and gastrointestinal tract of at least three lines of laboratory inbred mice for extended periods (3). The *N. musculi*-mouse model will allow studies of host association factors and their role in asymptomatic colonization of a natural host.

*N. musculi* was isolated from the oral cavity of wild-caught mice and stored as previously described (2). Strain NW831 was subcultured directly from the original stock of AP2031 (2). A cryopreserved stock was struck on GC medium base (GCB) agar with Kellogg’s supplements I and II and incubated at 37°C with 5% CO_2_ for 48 h (4). Isolated colonies were lawned on GCB agar. After 18 h, DNA was purified using an organic phenol-chloroform extraction method (5). DNA was sequenced using both Illumina and PacBio sequencing platforms. For Illumina sequencing, short-read libraries were generated with a KAPA HyperPrep kit and sequenced using 150-bp paired-end reads on the NovaSeq 6000 system. For PacBio sequencing, DNA was sheared using g-Tubes at 6,000 rpm, and libraries were constructed using the SMRTbell Express template prep kit 2.0. Libraries were size selected with Blue Pippin with a cutoff size of 7 kb and sequenced on the Sequel I system using Chemistry 3.0 (movie length, 1,200 min; read *N*_50_, 9,706 bp). PacBio reads were assembled into the main chromosome using SMRTLink 8.0/HGAP 4.0 with the microbial assembly module (the length_cutoff parameter was set to −1). Polishing was accomplished with the smrtlink-release_8.0 resequencing pipeline using the Arrow algorithm and then with Pilon 1.21 (6) using Illumina reads. The single contig obtained was trimmed of overlaps using Minimus 2 (7) and manually rotated to place the *dnaA* gene at nucleotide 4. Illumina reads were assembled separately using SPAdes 3.11.1 (8) to obtain the plasmid assembly. Default parameters were used for all software unless otherwise noted. Other features of the two sequencing methods are provided in Table 1.

**TABLE 1:** Further sequencing information for the *N. musculi* genome

Feature	Datum
Illumina sequencing	
SRA accession no.	SRX9090710
No. of reads	8,512,816
Coverage (×)	439
PacBio sequencing	
SRA accession no.	SRX9090709
No. of reads	209,605
Coverage (×)	603

The complete circular genome and plasmid were each assembled into single contigs with lengths of 2,928,421 bp (53% G+C content) and 4,650 bp (48% G+C content), respectively. DNA uptake sequences are hallmark repetitive elements in *Neisseria* species that facilitate neisserial transformation (9). There were 3,996 10-mer (GCCGTCTGAA) and 2,661 12-mer (AGGCCGTCTGAA) DNA uptake sequences in the genome and 0 in the plasmid. The genome and plasmid sequences were annotated using the Institute for Genome Sciences (IGS) annotation pipeline and by NCBI using PGAP 4.13 (10, 11). The IGS pipeline identified 2,787 genes (2,778 genes in the chromosome and 9 genes in the plasmid), 2,721 protein-coding sequences, 4 rRNA operons, and 54 tRNAs in the genome.

### Data availability.

The complete genome sequence of *N. musculi* has been deposited in GenBank under the accession numbers CP060414.1 (for the genome) and CP060415.1 (plasmid), BioProject number PRJNA656535, and BioSample number SAMN15791842.
